# Whole Genome Resequencing Reveals the Genetic Basis of Desert Arid Climate Adaptation in Lop Sheep

**DOI:** 10.3390/ani15182747

**Published:** 2025-09-19

**Authors:** Chenchen Yang, Changhai Gong, Abliz Khamili, Xiaopeng Li, Qifeng Gao, Hong Chen, Xin Xiang, Jieru Wang, Chunmei Han, Qinghua Gao

**Affiliations:** 1 College of Animal Science and Technology, Tarim University, Alar 843300, China; 10757223066@stumail.taru.edu.cn (C.Y.); miexiaochi@163.com (X.L.); xiang991201@163.com (X.X.); wjr1232023@126.com (J.W.); 2 School of Life Sciences, Henan University, Kaifeng 475000, China; 3 Xinjiang Bazhou Animal Husbandry Work Station, Bazhou 841000, China; 18999006858@163.com; 4 Bureau of Agriculture and Rural Development, Ruoqiang County, Bazhou 841000, China; 13779331376@163.com; 5 College of Life Science and Technology, Tarim University, Alar 843300, China; gqfsryd1415926@163.com (Q.G.); 107572024416@stumail.taru.edu.cn (H.C.); 6 Key Laboratory of Livestock and Forage Resources Utilization around Tarim, Ministry of Agriculture and Rural Areas, Alar 843300, China

**Keywords:** Lop sheep, desert arid climate, origin and evolution, environmental adaptation, selection signal

## Abstract

The Lop sheep (LOP), a unique local breed from Xinjiang, exhibits remarkable resilience to the harsh conditions of a desert arid climate and frequent sandstorms, alongside notable fecundity characteristics. This study aims to investigate the adaptability of LOP within this challenging environment by collecting whole blood samples from 110 LOP individuals in the Lop Nur region of Xinjiang for genome resequencing. The resulting data will be compared with whole genome resequencing information from 22 local sheep breeds worldwide to analyze the origin and evolution of LOP. Additionally, comparisons will be made with HUS sheep from warm and humid regions to identify genomic differences through selection signal analysis, thereby assessing the impact of a desert arid climate on the extreme living conditions of LOP. Finally, qPCR was used to preliminarily analyse the impact of the desert arid climate on the genome of the Bactrian sheep. Genetic diversity results indicate that LOP exhibits a relatively stable genetic structure alongside high genetic diversity. The results of population structure analysis and gene flow indicate that we can tentatively posit that LOP is a breed that originated from the Middle East, subsequently mixing with MGS upon its arrival in Xinjiang. Chinese local sheep breeds trace their origins to AMS, with the gene flow evolving from west to east, progressing through mountainous hills (BSBS), basins (LOP, HTS, CLHS, DLS), plains (MGS, TANS), and coastal areas (HUS). LOP is associated with ALTS, BSBS, HTS, CLHS, and DLS, as well as with MGS, HUS, TANS, WDS, and SSSP, in a context of gene exchange, with the degree of exchange diminishing in that order. Selection signal analysis revealed that the candidate genes identified are closely related to adaptation to desert arid climates and disease resistance (*PDGFD*, *NDUFS3*, *ATP1B2*, *ITGB8*, and *CD79A*), using HUS as the reference group. qPCR results demonstrated that LOP was significantly upregulated in cardiac, splenic, and lung tissues compared to HUS, suggesting that LOP plays a crucial role in cardiac function, immune response, and respiratory capacity.

## Simple Summary

The Lop sheep, a renowned local breed in China, is exceptionally well-adapted to desert pastures, exhibiting remarkable tolerance to extreme arid climates and roughage, along with strong resistance to environmental stresses. However, there is currently limited research on the population genetic structure and genetic relationships of Lop sheep. Furthermore, the extensive history of domestication and artificial selection over thousands of years has resulted in the emergence of distinct population genetic traits, yet the genetic basis underlying the genomes subject to selection remains unreported. In this study, we utilized whole genome resequencing data to analyze the genetic diversity, population structure, origin and evolution, and selection signals of Lop sheep. Our objective is to systematically explore the genetic structure and composition of the Lop sheep population and to identify the genomic regions that have been subjected to selection throughout the process of selective domestication. This research aims to comprehensively evaluate the value of the genetic resources of Lop sheep and provide a theoretical foundation for the conservation of these genetic resources, breed selection, and future genetic improvement efforts.

## 1. Introduction

Lop sheep (LOP) inhabit the southeastern edge of the Taklimakan Desert and the Lop Nor region, which is characterized by a typical temperate continental desert climate. This region experiences extremely dry and hot conditions, with summer temperatures exceeding 40 °C and winter temperatures dropping below −20 °C. The annual precipitation is approximately 20 mm, while evaporation rates exceed 3000 mm. The area enjoys over 3200 h of sunshine annually, and there is a significant temperature difference between day and night, with an annual cumulative temperature exceeding 4500 °C. Wind erosion is prevalent, particularly during the windy seasons from March to May and June to August, with more than 60 days classified as class 8 gale days. This often results in sandstorm conditions, and the region experiences between 115 and 193 days of floating dust weather each year [1].

LOP is a unique local sheep breed highly adapted to desert pastures, characterized by its resilience to extreme arid climates, tolerance for roughage, and robust resistance to adversities. This breed is primarily found in Ruoqiang and Yuli counties within the Bayin’guoleng Mongol Autonomous Prefecture of Xinjiang. Previous studies indicated that the LOP populations in Ruoqiang (LOPRQ) and Yuli (LOPYL) share a common lineage; however, there is currently some genetic differentiation between these two populations [2]. The vast expanse of the Taklamakan Desert region and the significant distance between human settlements have created natural geographical barriers, which have impeded gene exchange between LOP and other sheep breeds for an extended period [3]. As a result, LOP has managed to reproduce and preserve its unique traits. The breed has undergone greater natural selection pressures compared to artificial selection, leading to its exceptional survival capabilities and the development of specialized body structures and physiological mechanisms that facilitate adaptations to desert environments, including disease resistance, UV resistance, and heat tolerance.

In the process of natural selection and artificial breeding, sheep adapt to various ecological environments through local genetic variations [4,5]. Currently, whole genome sequencing (WGS) has increasingly focused on the study of local breeds exhibiting distinct adaptive traits, such as disease resistance (*GPR35*, *SH2B2*) [6], altitude adaptation (*PAPSS2* and *RXFP2*), horn development (*RXFP2*) [7], fleece characteristics (*PDE4B*, *ABCD4*), growth and development (*HMGA2*, *MSRB3*), and reproduction (*BVES*, *WNT11*) [8]. Zhong demonstrated the diverse breeding histories of three local sheep branches in China (Mongolian, Kazakh, and Tibetan) and provided insights into the genetic structure of these populations under varying geographic distributions and ecological factors [9]. Zhang [10] utilized 14 sheep breeds from Xinjiang to identify candidate genes associated with lung diseases in sheep, including *GZMK*, *IL1A*, *IL1B*, *FGFR2*, *MS4A1*, and *LEF-1*. Jin [11] screened candidate genes related to the selection of sheep in arid regions, identifying genes such as *TBXT*, *TSHR*, *ABCD4*, and *TEX11*, based on 41 representative local sheep breeds in China. Wang [12] investigated local sheep in the northeastern Tarim Basin to identify candidate genes linked to adaptation in desert environments, including *SOD1*, *TSHR*, and *FOXA2*. These findings can serve as a reference for the genetic breeding of local sheep breeds in China.

This study systematically analysed the population structure and origin differentiation of LOP sheep alongside 22 domestic and wild sheep populations worldwide, based on whole genome resequencing data. It precisely identified several single nucleotide polymorphisms (SNPs) significantly associated with adaptations to extreme environments such as desert aridity and disease resistance. The findings not only provide key targets for molecular breeding of drought-tolerant sheep but also establish a theoretical foundation for optimizing strategies to utilize and conserve China’s indigenous sheep genetic resources. By integrating gene pool selection with rapid propagation techniques, it is possible to significantly enhance the stress resistance and production performance of desert sheep while further strengthening the genetic diversity of local breeds. This approach facilitates the sustainable utilization of distinctive resources and promotes high-quality industrial development.

## 2. Materials and Methods

### 2.1. Animal Care

All animal experiments were conducted in accordance with the “Regulations and Guidelines for the Management of Experimental Animals” established by the Ministry of Science and Technology (Beijing, China, 2020 revision). This study was approved by the Institutional Animal Care and Use Committee of Tarim University, Xinjiang, China (SYXK2020-009).

### 2.2. Sample Collection and Sequencing

The Ruoqiang County Lop sheep are mainly grazed primitively, and the Yuli County Lop sheep are mainly farmed on a large scale. In this study, jugular vein blood samples were collected from 80 and 30 Lop sheep from Ruoqiang and Yuli Counties, respectively, in Bayin’guoleng Autonomous Prefecture, Xinjiang, China (Supplementary Table S1). The Ruoqiang County Lop sheep ware mainly grazed primitively, and the Yuli County Lop sheep ware mainly farmed on a large scale. No experimental animals were slaughtered during the sampling process. Whole genome second-generation sequencing was performed at a depth of 10X, with library preparation and sequencing completed according to the manufacturer’s recommended workflow (https://www.bgi.com/, accessed on 20 July 2022). Additionally, datasets for 22 other sheep breeds were obtained from the National Centre for Biotechnology Information (NCBI) (https://www.ncbi.nlm.nih.gov/, accessed on 25 July 2022) (Supplementary Table S2).

### 2.3. Genotyping and Quality Control

Quality control was performed on raw data using Fastq v0.23.2 [13] with default parameters; bwa-mem2 v2.2.1 [14] was employed to align the quality-controlled data against the sheep reference genome (NCBI: GCF_016772045.1); SAMtools v.1.9 [15] was used for file conversion, depth detection, data filtering, and sorting; GATK v4.1.4.1 [16] was used for deduplication, data merging, quality control (QD < 2.0||MQ < 40.0||FS > 60.0||MQRankSum < −12.5||ReadPosRankSum < −8.0||SOR > 3.0), and variant detection; and final quality control was performed using VCFtools v0.1.16 [17] and PLINK v1.90 (https://www.cog-genomics.org/plink/, accessed on 25 March 2025) (-hwe 1 × 10^−7^ -maf 0.05).

### 2.4. Genetic Diversity and Population Structure

PLINK was used to calculate genetic diversity parameters, including minor allele frequency (MAF), runs of homozygosity-based inbreeding coefficient (Froh), observed heterozygosity (Ho), and expected heterozygosity (He). PopLDdecay v3.40 software (https://github.com/BGI-shenzhen/PopLDdecay.git, accessed on 3 April 2025) was employed for linkage disequilibrium analysis. Principal component analysis (PCA) was performed using PLINK; a neighbor-joining tree (NJtree) was constructed with VCF2Dis (https://github.com/BGI-shenzhen/VCF2Dis, accessed on 10 April 2025); and Admixture software (https://dalexander.github.io/admixture/binaries/admixture_linux-1.3.0.tar.gz, accessed on 15 April 2025) was utilized for ancestral component analysis. Gene flow analysis was performed using Treemix v1.13 software [18] and Dsuite v0.5 software [19]. The outgroup used in the D-test was AMS, P2 was ALTS, and P3 comprised BSBS, CLHS, DLS, HTS, and LOP.

### 2.5. Selection of Signal Analyses

To investigate the adaptive genetic mechanisms of argali sheep in extreme arid environments, we employed both Fixation Index (FST) and π ratio methods to analyse selection signals.

The genetic fixation index (FST) is a commonly employed method for analyzing selection signals [20]. The calculation formula is as follows:

$$FST=\frac{MSP-MSG}{MSP+(nc-1)MSG}$$

MSG stands for the mean square error within populations, MSP stands for the mean square error between populations, and nc denotes the corrected average sample size for the entire population.

Nucleotide polymorphism utilizes π as a key parameter. This approach was introduced to calculate the reduction in population polymorphism of domesticated populations in contrast to wild ones [21]. The formula is as follows:

$$\pi \;ratio=\frac{\pi (wild)}{\pi (domesticated)}$$

The π ratio refers to the ratio of nucleotide diversity between wild and domesticated populations, where π(wild) represents the nucleotide diversity of wild populations and π(domesticated) stands for that of domesticated populations. When Log_2_(π ratio) yields positive values, it signifies reduced nucleotide polymorphism within the window, a sign of selection during the domestication process.

Under autosomal settings, VCFtools software was utilized to calculate the FST and π ratio. A sliding window strategy was adopted here, with the window size set to 100 kb and the step size at 50 kb. Afterward, a top 1% threshold was applied to screen out significant selection signals, followed by filtering to obtain intersecting genes.

### 2.6. Gene Functional Enrichment Analysis

Annotate genes from the intersection of the FST and π ratio (GCF_016772045.1.gtf); perform functional and pathway enrichment analysis on candidate genes using Gene Ontology (GO) and the Kyoto Encyclopedia of Genes and Genomes (KEGG) [22,23]. Plotting was performed using R v4.4.3 software (https://www.r-project.org/, accessed on 2 May 2025) and WSX (http://www.bioinformatics.com.cn/, accessed on 12 May 2025).

### 2.7. Methods for Candidate Gene Validation

Samples were collected from the LOP preservation farm and the HUS farm located in Ruoqiang County, Bayin’guoleng Autonomous Prefecture, Xinjiang, China. Six LOP and HUS samples were randomly selected, and organ tissues from the heart, liver, spleen, lung, and kidney were harvested from both LOP and HUS. These samples were labeled and stored in liquid nitrogen for future analysis. The reagents and instruments utilized in the experiments are detailed in Supplementary File S3.

The total RNA extraction process involved heart, liver, spleen, lung, and kidney tissues from LOP and HUS, followed by reverse transcription to synthesize the first strand of cDNA, and primer design for five candidate genes (Table 1). For further information, please refer to Supplementary File S3.

## 3. Results

### 3.1. Genetic Diversity

The results of genetic diversity analysis conducted on 283 sheep from 23 breeds are presented in Table 1. The minor allele frequency (MAF) of Xinjiang local sheep breeds ranged from 0.1992 (DLS) to 0.2325 (LOP), while the observed heterozygosity (Ho) ranged from 0.2515 (HTS) to 0.2904 (LOP), and the expected heterozygosity (He) varied from 0.2607 (DLS) to 0.2994 (LOP). In contrast, the MAF of other regional sheep breeds ranged from 0.1879 (MLNS) to 0.2065 (XWHS), the Ho varied from 0.1994 (Ouessant) to 0.2929 (XWHS), and the He ranged from 0.1920 (Ouessant) to 0.2689 (XWHS). The results indicate that the He of LOP is the highest among all varieties, suggesting that its genetic diversity is superior compared to the others. Although the observed heterozygosity (0.2904) in Lop sheep was slightly lower than the expected heterozygosity (0.2994), its He value remained significantly higher than all control groups, indicating that the breed retains a rich allele pool. The marginally lower Ho value compared to He reflects mild inbreeding within the desert-confined Lop sheep population (Table 2).

### 3.2. Population Structure and Gene Flow

**Figure 1:**
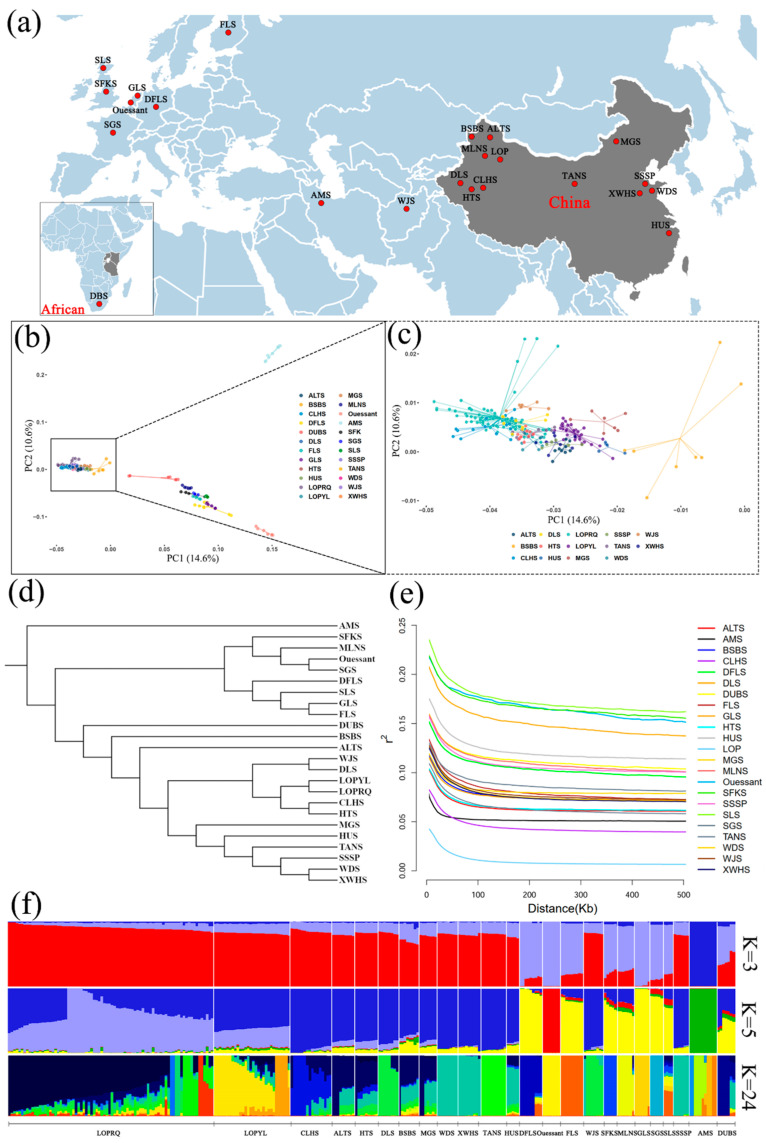
Results of population genetic structure analysis of 23 sheep breeds. (**a**) Geographic distribution of 23 local sheep breeds. (**b**,**c**) Results of PCA of 23 local sheep breeds. (**d**) Results of phylogenetic tree of 23 local sheep breeds. (**e**) Analysis of LD linkage disequilibrium of 23 local sheep breeds. (**f**) Admixture results of 23 local sheep breeds, with different colors indicating distinct ancestral components.

Through principal component analysis (PCA), NJtree, and ADMIXTURE analyses, we comprehensively elucidated the results of the population genetic relationship analyses between the LOP population and 23 local sheep breeds (Figure 1).

The PCA results indicated that the AMS, local sheep from Western European countries, and Chinese local sheep breeds were effectively distinguished among the resequencing data of 23 sheep breeds worldwide (Figure 1b). Notably, the Chinese local sheep exhibited a mosaic distribution due to their extensive geographical range and diverse ecological environments. The Ruoqiang and Yuli LOPs were identified as distinct sub-branches, implying that the LOP populations in these two regions are currently differentiated. However, the genetic distance between the Ruoqiang and Yuli LOP populations is relatively close, placing them within a larger branch. Whole genome resequencing analysis confirms that the Ruoqiang and Yuli LOPs share a common lineage (Figure 1a,b).

The NJtree results indicate that the LOP breed forms an independent branch distinct from other sheep breeds worldwide. Furthermore, the Yuli and Ruoqiang LOP breeds exhibit no intermixing, instead clustering into separate sub-branches. The LOP breed shows a close genetic relationship with DLS, CLHS, HTS, and WJS. In China, local sheep breeds can be categorized into three major branches, which include AMS and local breeds from Western European countries. The genetic diversity among all sheep breeds is closely associated with geographical distance. Notably, the LOP breed is among the younger local sheep breeds in Xinjiang, while the bloodlines of BSBS, ALTS, and DLS are comparatively older within this region (Figure 1c,d).

The results of the PopLDdecay analysis indicated that the linkage disequilibrium (LD) decay trend across 23 sheep breeds decreased with increasing marker spacing. However, there were significant differences in the extent of decline among the breeds. Notably, the LOP breed exhibited the fastest LD decay, while the SLS breed demonstrated the slowest decay at the same marker spacing. This variation is illustrated in (Figure 1e).

The ADMIXTURE analysis revealed that at K = 3, the AMS population was distinctly separated from other sheep bloodlines. At K = 5, both Ruoqiang and Yuli LOP breeds maintained their unique genetic identities while showing genetic relatedness to Xinjiang local sheep. The sheep populations were categorized into three groups: AMS, Western European local sheep, and Chinese local sheep. This classification aligns with the findings from the PCA and NJtree analyses (Figure 1f).

**Figure 2:**
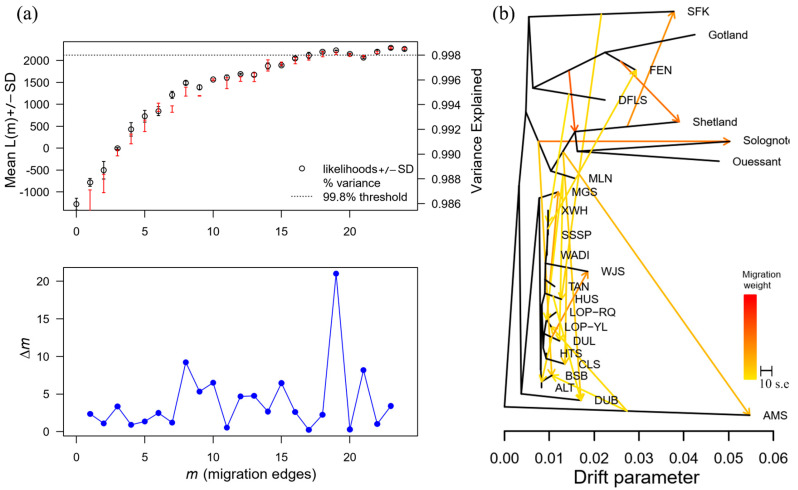
TreeMix analysis of 23 sheep breeds. (**a**) OptM best fit plot evaluating different m results for TreeMix, with the best fit at *m* = 19. (**b**) Results of TreeMix analyses highlighting the direction and extent of the likelihood tree and gene penetration for all breeds.

Using TreeMix to construct the maximum likelihood tree for all populations, this method not only reveals population differentiation events but also effectively captures gene flow or migration events. According to the OptM results: when AMS is used as the outgroup, the optimal value is m = 19 (Figure 2a); gene flow occurs between AMS and local sheep breeds in China and Western European countries. Gene exchange events primarily occur among local sheep breeds in China, with MLNS infiltrating LOP and DLS, TANS infiltrating MGS, DLS infiltrating WJS, HUS infiltrating DUBS, and AMS infiltrating LOPYL (Figure 2b) (Supplementary File S4).

**Figure 3:**
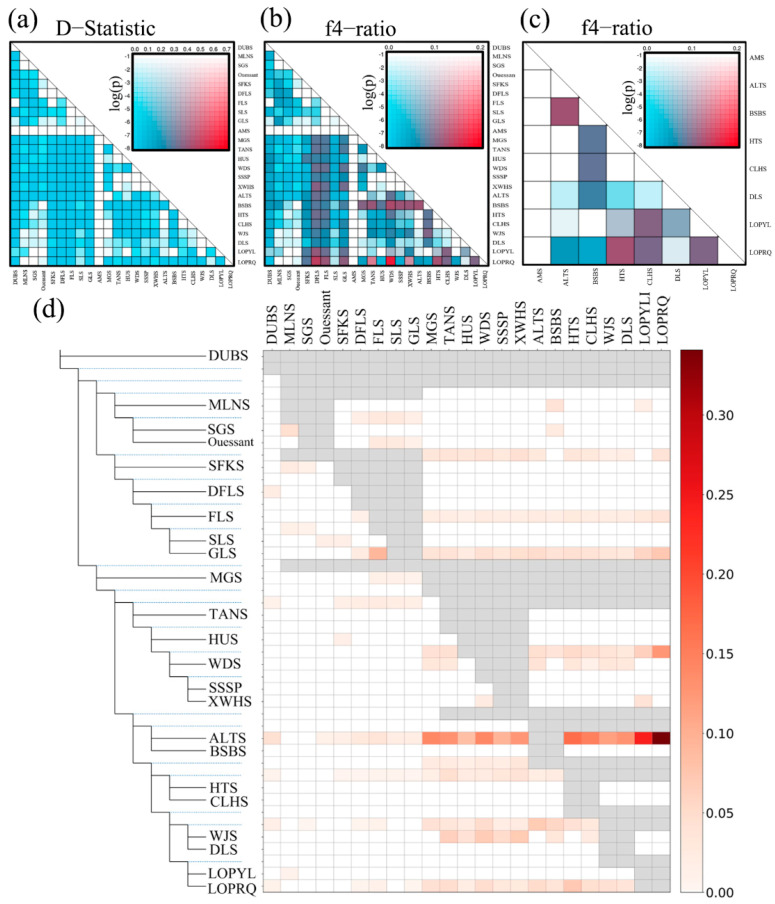
Probing gene flow between species using the ABBA-BABA method. (**a**) Heatmap of the D-statistic test with AMS as the outgroup, showing the D-statistic results and their significance for each pair of species. (**b**) Heatmap of the f4 ratio statistical test with AMS as the outgroup, highlighting the f4 ratio results and their significance for each pair of species. (**c**) Heatmap of the f4 ratio statistical test within Xinjiang indigenous sheep populations (with AMS as the outgroup), revealing the f4 ratio results and their significance for each pair of species. Red and blue represent high and low D-statistic values, respectively; the color intensity corresponds to the *p*-value (on a logarithmic scale), and the display order of species has been rearranged. (**d**) F-Branch metric matrix, showing the gene flow of 23 breeds in evolutionary branches and terminal branches.

**Figure 4:**
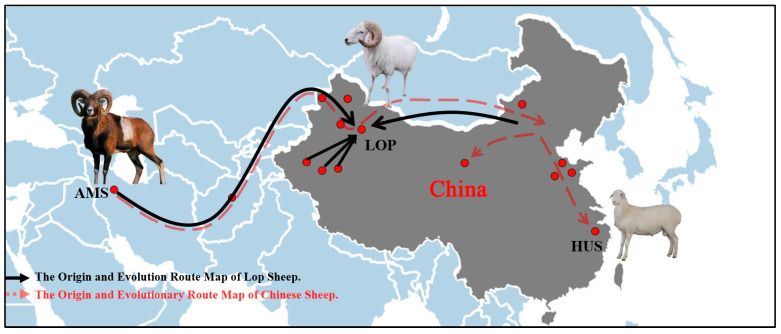
The origin and evolutionary route map of Lop sheep and Chinese indigenous sheep.

To further explore the phenomenon of gene infiltration across various sheep breeds, we conducted an ABBA-BABA analysis. The results indicated that when AMS was designated as P1, extensive gene exchange occurred between ALTS and several local Chinese sheep breeds, including LOP, MGS, TANS, HUS, WDS, SSSP, BSBS, HTS, CLHS, and DLS (Figure 3) In conjunction with the TreeMix results, it was observed that gene exchanges among local sheep breeds in China originated from AMS. Furthermore, the gene flow in Chinese sheep breeds evolved from the west to the east, progressively moving towards mountainous hills (BSBS), basins (LOP, HTS, CLHS, WJS, DLS), plains (MGS, TANS), and coastal areas (HUS) (Figure 4) (Supplementary File S5).

### 3.3. Selective Signal Analysis

**Figure 5:**
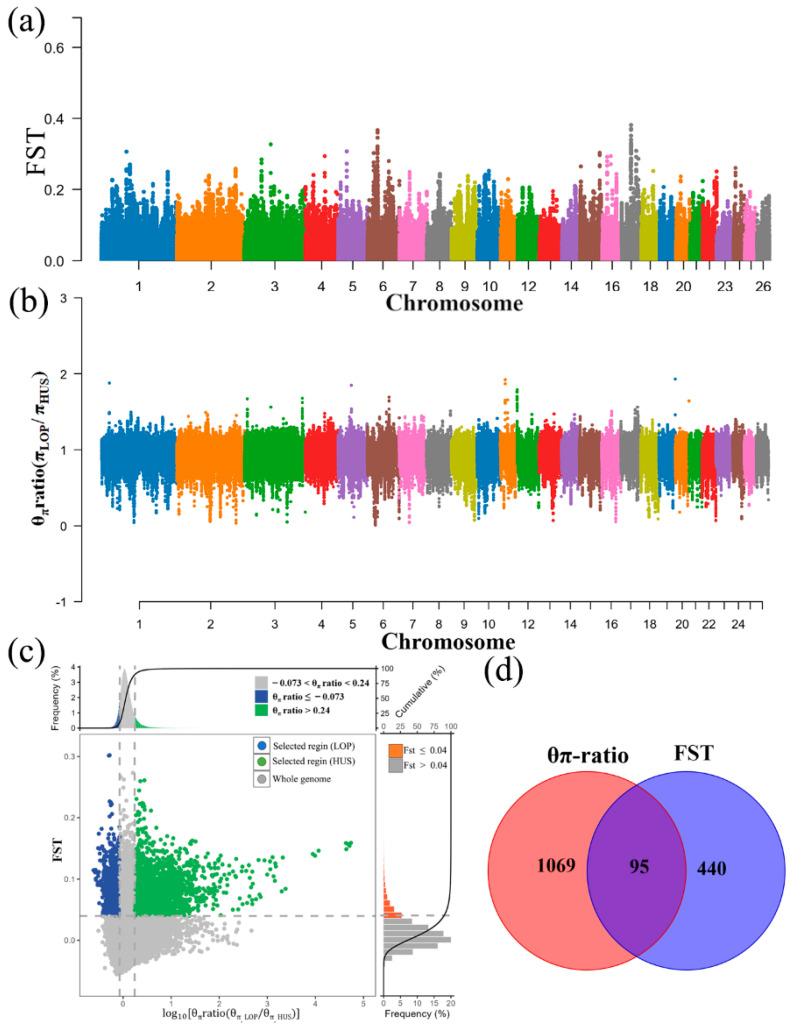
Selection signal analysis of LOP and HUS. (**a**) FST Manhattan plot results for LOP and HUS. (**b**) π ratio Manhattan plot results for LOP and HUS. (**c**) Selection elimination analysis results based on FST and π ratio. (**d**) Venn diagram results showing the intersection of FST and π ratio findings.

In this experiment, we analyzed the genetic mechanisms underlying adaptations such as heat tolerance and robust resistance of LOP in extreme desert environments, based on annual rainfall data from the last 20 years (LOP: drought < 50 mm). The results indicated that a total of 535 and 1164 candidate genes were identified after screening using the FST (0.136) and π ratio (1.205) methods, with HUS serving as the reference population and the top 1% designated as the candidate region (Figure 5a–c). After removing duplicate and invalid genes, we took the intersection of the candidate genes identified by the FST and π ratio, resulting in a total of 95 candidate genes (Figure 5d).

**Figure 6:**
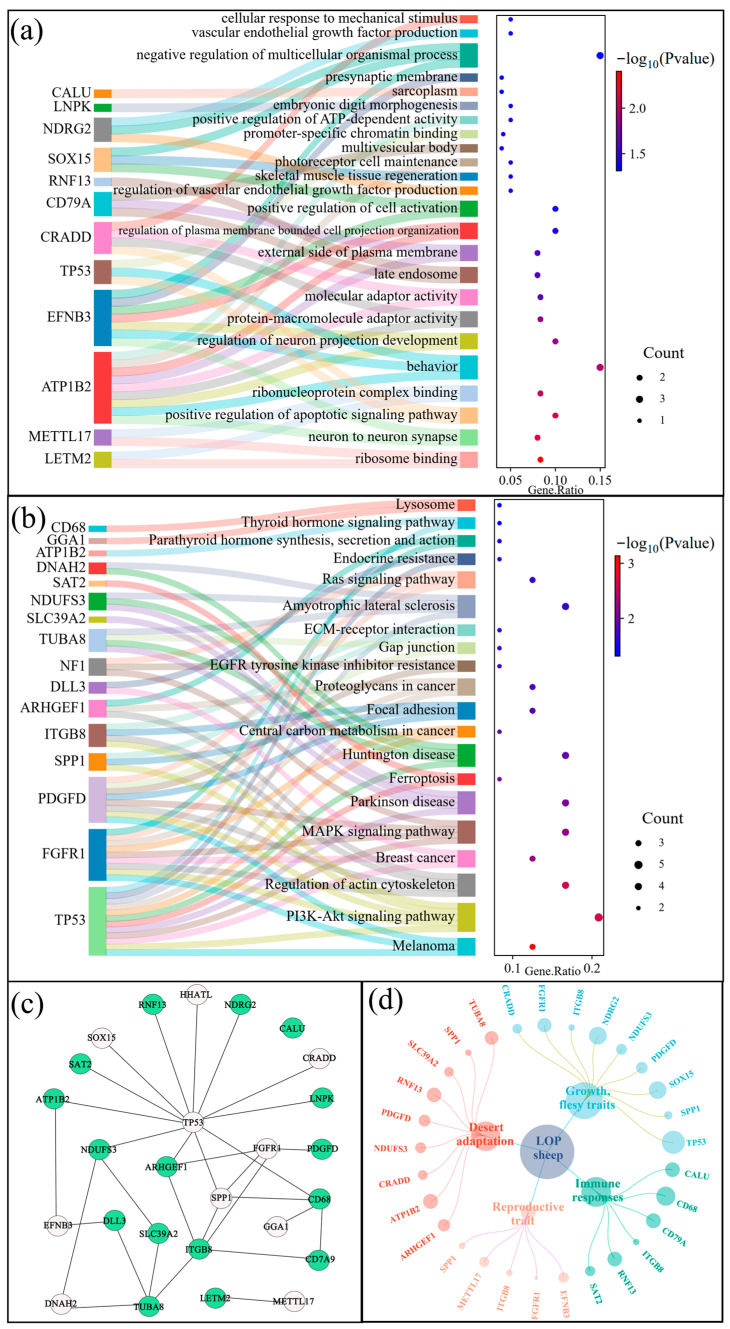
GO and KEGG pathway enrichment analysis. (**a**) The Sankey-Dot plot of the Top 24 GO terms. (**b**) This Sankey-Dot graph shows 20 key KEGG pathways. (**c**). Gene networking analysis of 26 overlapping genes (*p* < 0.05); green is a candidate gene for immune response in Lop sheep. (**d**) Network aggregation map of 26 candidate genes annotated to the five major traits.

A total of 32 and 21 functions and pathways were enriched through Gene Ontology (GO) and Kyoto Encyclopedia of Genes and Genomes (KEGG) enrichment analyses (*p* < 0.05) (Figure 6a,b). The enriched functions and pathways were associated with behavior (e.g., GO:0007610), immunity (e.g., oas04151, PI3K-Akt signaling pathway; oas04510, Focal adhesion), cell signaling (e.g., GO:0030674, protein–macromolecule adaptor activity), and developmental processes (e.g., GO:0010574, regulation of vascular endothelial growth factor production; GO:0051241, negative regulation of multicellular organismal process), among others (Figure 6a,b). For the screened candidate genes, enrichment analyses and literature searches indicated that they were linked to the adaptation of LOP in extreme arid environments. Specifically, genes such as *NDUFS3*, *SLC39A2*, *ATP1B2*, *CRADD*, *RNF13*, *SPP1*, *ARHGEF1*, *PDGFD*, and *TUBAD* were associated with heat resistance, thermal insulation, UV resistance, and resilience to harsh environments. Additionally, genes *ITGB8*, *CD79A*, *CALU*, *RNF13*, *SAT2*, and *CD68* were found to be related to immune responses (Figure 6c,d) (Supplementary Table S6).

### 3.4. Candidate Gene Validation

**Figure 7:**
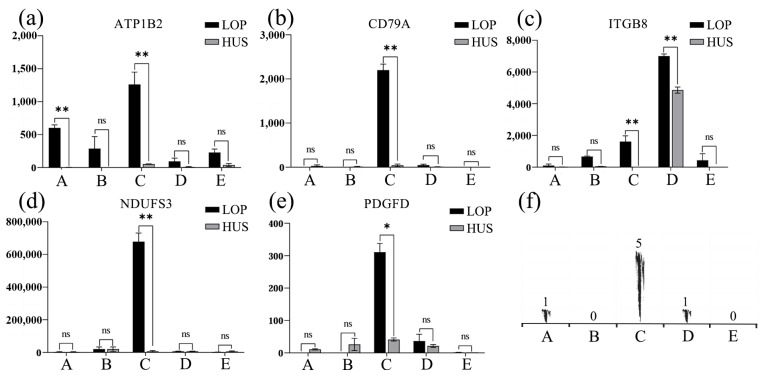
The expression levels of the five candidate genes (a–e) were analyzed in various organs of LOP and HUS, specifically the heart (**a**), liver (**b**), spleen (**c**), lung (**d**), and kidney (**e**). (**f**) The statistical significance of these expressions across the five organs is denoted, where ‘**’ indicates highly significant results, ‘*’denotes significant results, and ‘ns’ signifies non-significant findings.

Quantitative PCR (qPCR) analysis of five candidate genes (*PDGFD*, *NDUFS3*, *ATP1B2*, *ITGB8*, and *CD79A*) revealed significant expression levels in the heart, liver, spleen, and lung tissues of Lop sheep. Notably, *ATP1B2* exhibited a highly significant expression in the heart of Lop sheep (*p* < 0.01). Additionally, *ATP1B2*, *CD79A*, *NDUFS3*, and *ITGB8* demonstrated highly significant expression in the spleen (*p* < 0.01), while *PDGFD* was significantly expressed in the spleen (*p* < 0.05). Furthermore, *ITGB8* showed highly significant expression in the lungs of Lop sheep (*p* < 0.01) (Figure 7).

## 4. Discussion

At the genome-wide level, the minor allele frequency (MAF), observed heterozygosity (Ho), and expected heterozygosity (He) values of Xinjiang local sheep breeds were generally higher than those of other sheep breeds, indicating that the genetic diversity of Xinjiang local sheep breeds is richer than that of other breeds. Among these, the genetic diversity of the LOP breed was found to be higher than that of other Xinjiang local sheep breeds. This may be attributed to the LOP’s long-term adaptation to the Lop area, which is characterized by extreme environmental conditions, including high temperatures, drought, and saline soil. LOP has undergone strong natural selection pressures, where only individuals that can adapt to and tolerate these harsh conditions survive and reproduce.

LD decay, from the perspective of Chinese local sheep breeds, shows a more rapid trend among breeds inhabiting Xinjiang’s relatively harsh natural environment (LOP, CLHS, ALT, HTS, BSBS, and DLS), with LOP exhibiting the fastest rate of decay. Sheep breeds living in Xinjiang’s relatively harsh natural environment face prolonged exposure to adverse conditions such as intense heat and drought. Under such conditions, natural selection exerts a stronger influence, leading to the rapid fixation of genetic loci associated with adaptation to harsh environments. For instance, genes linked to traits such as cold resistance, drought tolerance, and efficient utilization of limited food resources spread rapidly within populations under natural selection, resulting in accelerated LD decay in the relevant chromosomal regions. In contrast, breeds inhabiting regions abundant in green fodder resources (MGS, WDS, TANS, XWHS, and HT) exhibit a slower LD decay trend. These areas offer relatively favorable environmental conditions, where sheep face lesser survival pressures. Consequently, natural selection exerts less intense selection pressure on specific adaptive genes, resulting in slower diffusion and fixation of these genes within the population, hence the slower LD decay.

The results of the population structure analysis revealed a distinct separation between the LOPYL and LOPRQ populations. This separation may be attributed to the environmental differences between Ruoqiang County, characterized by desert and semi-desert pastures, and Yuli County, which enjoys a more moderate and humid climate. The LOPYL population has been isolated for an extended period due to its habitation in the vast desert region, while the LOPRQ population has historically resided in mountainous areas with primitive grazing practices, which hinder transportation and led to prolonged isolation between the two populations.

Consequently, this isolation has resulted in insufficient genetic exchange and subsequent genetic differentiation between them, making the two populations genetically distinct. Additionally, analysis using the Treemix and ABBA-BABA methods indicated that Chinese local sheep have undergone genetic exchange with AMS and local sheep from Western European countries, with the highest exchange observed between AMS and Xinjiang local sheep. There was significant gene flow among the local sheep breeds in Xinjiang (ALTS, BSBS, HTS, LOP, CLHS, DLS), which have continued to differentiate due to human migratory activities, resulting in the formation of several desert-adapted sheep breeds. Furthermore, gene flow was also noted between ALTS and other local sheep breeds in China (ALTS, MGS, TANS, WDS, HUS, SSSP, XWHS). Therefore, we propose that the ancestor of Chinese local sheep is the AMS lineage. This conclusion aligns with findings from whole genome resequencing studies on gene flow in both wild and domesticated sheep globally [24].

LOP is distributed across the Taklamakan Desert and the Lop Nor region, where summer temperatures exceed 40 °C and winter temperatures drop below −20 °C, resulting in significant diurnal temperature variation in the desert. LOP inhabits altitudes ranging from 800 to 6900 m, whereas HUS is found along the Taihu Plain in China, averaging approximately 300 m above sea level. This ecological context imposes exceptionally high demands on LOP’s heat and cold tolerance.

Candidate genes associated with local adaptation in extreme arid environments were identified through Gene Ontology (GO) and Kyoto Encyclopedia of Genes and Genomes (KEGG) enrichment analyses. Notable genes include *NDUFS3*, *SLC39A2*, *ATP1B2*, *CRADD*, *RNF13*, *SPP1*, *ARHGEF1*, *PDGFD*, and *TUBAD.* The *NDUFS3* gene has been linked to heat tolerance in African native cattle, influencing the capacity of brown adipose tissue to generate heat for thermoregulation [25,26]. Genome-wide analyses that elucidate bovine population structure and genetic infiltration have highlighted *SLC39A2*’s significant role in vascularity, erythropoiesis, and cardiac function under hypoxic conditions, as well as its involvement in skin growth and wound healing [27,28]. *ATP1B2* has been associated with myocardial contraction in response to hypoxia, serving as a genetic marker for heat tolerance traits in Chinese fine-wooled sheep and Holstein cows [29,30]. *CRADD* is linked to adaptations in the hot, arid, and hostile environments experienced by the Chinese endemic breed, Nyala chicken [31]. In extreme desert conditions, *RNF13* may facilitate the adaptation of LOP to harsh environments by modulating cellular stress responses and preserving cellular homeostasis [32]. *SPP1* has been associated with environmental resistance in Runs of Homozygosity (ROH) analyses of local Croatian sheep breeds [33]. *ARHGEF1* has been identified as a contributor to thermal insulation in cold environments [34]. *PDGFD* has been proposed as a candidate gene for adaptation in high-altitude sheep, closely associated with tail type and lipid metabolism in local Xinjiang sheep breeds, and confirmed to influence fat deposition in indigenous Chinese extreme-tailed sheep [35,36]. The *TUBA8* gene, which regulates the cytoskeleton, has been found to enhance skeletal traits in the adaptive evolution of the Jinchuan yak population in extreme plateau environments and is also a potential candidate for the multi-ribbed trait [37]. Through the synergistic action of these genes, LOP can better adapt to extreme arid environments and enhance its resistance to heat, drought, and diseases.

Located in the eastern part of the Tarim Basin in Xinjiang, China, and at the easternmost edge of the Taklamakan Desert, the largest desert in China, Lop is a world-renowned arid center characterized by a windy season lasting for half the year, which frequently leads to sandstorms. Annually, there are between 115 and 193 days of dusty weather, contributing to LOP’s remarkable resistance to pneumonia under these climatic conditions. The local herders primarily engage in primitive grazing, subjecting LOP to significant natural selection pressures. Consequently, LOP exhibits strong disease resistance and environmental adaptability. Through GO and KEGG enrichment analysis, genes such as *ITGB8*, *CD79A*, *CALU*, *RNF13*, *SAT2*, and *CD68* have been identified as being associated with disease resistance. *ITGB8* serves as a key mediator in the early inflammatory response during respiratory epithelial inflammation, promoting the expression of transforming growth factor-β1 (TGF-β1) and the accumulation of regulatory T lymphocytes (Treg), thereby enhancing the regression of lung inflammation [38]. Furthermore, *ITGB8* has been linked to cashmere formation and development in sheep in cold environments [39]. *CD79A* has been shown to play a crucial role in B cell-mediated immune responses, particularly regarding the regulation of lung inflammation and immune cell activation during Mycoplasma pneumoniae infections in sheep [40]. *CALU* may be involved in regulating intracellular calcium homeostasis, which is essential for maintaining mucosal cilia clearance functions. The downregulation of *RNF13* may correlate with the temporary suppression of retinal pigment epithelial (RPE) function and the initiation of immune responses, both of which are critical for cellular function and immune regulation following retinal detachment [41]. The upregulation of *CD68* is associated with increased myocardial inflammation and fibrosis, indicating its significant role in maternal obesity-induced cardiovascular disease in offspring, particularly during macrophage-mediated inflammatory responses and tissue damage [42,43]. This immune dominance results from long-term natural selection and evolution, which ensures the survival and reproduction of LOP in harsh environments.

**Figure 8:**
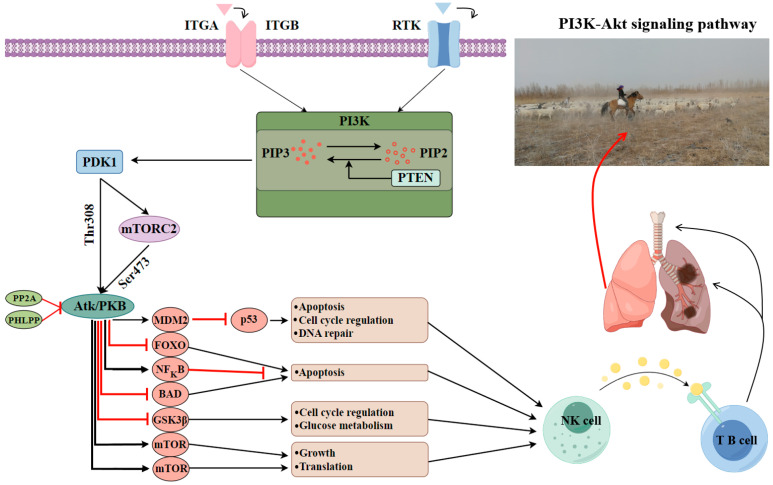
The figure explains the PI3K-Akt signaling pathway, which improves resistance to lung disease in Lop sheep by regulating processes such as immune response, inflammatory response, cell survival, and migration.

The *ATP1B2* gene exhibits significantly elevated expression levels in both the heart and spleen, indicating its crucial role in the maintenance of myocardial contractile function and immune response in LOP. This finding aligns with the observed myocardial contraction in response to hypoxia, a trait characteristic of Chinese fine-wooled sheep. Additionally, the expression changes of this gene in the HPA axis of pigs correlate with stress responses and behavioral performance, suggesting its potential role in regulating stress responses [44]. Furthermore, the methylation status of the *ATP1B2* gene is associated with mechanisms of tumor growth inhibition [45]. The *CD79A* gene, which is also highly expressed in the spleen of Lop sheep, is vital for B cells as part of the B-cell receptor (BCR) complex, facilitating signal transduction and B-cell development, particularly in regulating lung inflammation and immune activation [46]. The *ITGB8* gene shows significant expression in the lungs and spleen of LOP, playing a key role in myeloid phagocytosis of apoptotic cells in the lungs of mice. It enhances the migratory and immunomodulatory capacities of myeloid phagocytes through increased expression, leading to Treg accumulation and ultimately promoting the resolution of acute inflammation [47]. Lastly, the *NDUFS3* gene, significantly expressed in the spleen of LOP, protects the kidneys from sepsis-induced acute kidney injury (SI-AKI) by inhibiting ferroptosis and mitochondrial damage via the activation of the AMPK pathway [48]. The *PDGFD* gene, significantly expressed in the spleen of LOP, plays a crucial role in kidney protection during ocular diseases by upregulating immune proteasome genes. This upregulation increases intercellular interactions and promotes epithelial–mesenchymal transition (EMT) in retinal pigment epithelium (RPE) cells. Consequently, this effect not only enhances the antigen processing and presentation capacity of RPE cells but also stimulates angiogenesis [49]. Furthermore, both the *ITGB8* and *PDGFD* genes are regulated by the PI3K-Akt signaling pathway, with the *ITGB8* gene acting as an upstream regulator [50]. Based on the results of our qPCR analysis, we conclude that the PI3K-Akt signaling pathway may be pivotal in modulating the immune response of LOP, aiding in the resistance against pathogens and the maintenance of normal lung function under the extreme arid conditions characteristic of the perennially windy and sandy Lop region (Figure 8).

## 5. Conclusions

By comparing the whole genome resequencing data of 23 local sheep breeds worldwide, we found that LOP exhibits rich genetic diversity. Chinese local sheep breeds are believed to have originated from AMS, with gene exchanges occurring with ALTS. We hypothesize that LOP is a breed that first arrived in Xinjiang from the Middle East and subsequently mixed with Mongolian sheep. Through qPCR analysis of five candidate genes in five organ tissues of LOP and HUS, we discovered that LOP exhibited significant expression of several genes in the heart, spleen, and lungs. This suggests that LOP may possess enhanced capabilities in cardiac function, immune response, and respiratory function.

## Figures and Tables

**Table 1:** Amplification primer information.

Primer Name	Primer Sequence	Annealing Temperature	Fragment Length
*PDGFD*	F: CCAACCTCAGGCGAGATGAG	57	152
	R: TGTGAGGTGATTGCTCTCAAGT		
*NDUFS3*	F: GGTATGAGAGGGAGATCTGGGA	59	133
	R: TCAACATAGCCAGACAGCGG		
*ATP1B2*	F: ACCAGGGTTGATGATTCGCC	57	124
	R: GGAGTCATTGTAAGGCTCCAAG		
*ITGB8*	F: GCGGACTGCTTTGCATTATGT	57	161
	R: TGCACATCTGTTGTCTTCACTT		
*CD79A*	F: GAGAAGATGCCTGAGGGTCC	59	96
	R: CCATGAGGAGTTGCCCAGG		

**Table 2:** The results of genetic diversity in 23 sheep breeds.

Region	Breed	MAF	Ho	He
China	ALTS	0.2050	0.2573	0.2674
	BSBS	0.2078	0.2817	0.2707
	CLHS	0.2058	0.2617	0.2699
	DLS	0.1992	0.2665	0.2607
	HTS	0.2055	0.2515	0.2679
	WDS	0.2029	0.2785	0.2645
	LOP	0.2325	0.2904	0.2994
Other countries	AMS	0.1867	0.2445	0.2464
	DFLS	0.1755	0.2526	0.2324
	DUBS	0.1860	0.2687	0.2444
	FLS	0.1904	0.2716	0.2502
	GLS	0.1599	0.2362	0.2092
	HUS	0.1977	0.2879	0.2565
	MGS	0.2024	0.2569	0.2652
	MLNS	0.1879	0.2812	0.2458
	Ouessant	0.1454	0.1994	0.1920
	SFKS	0.1675	0.2612	0.2184
	SLS	0.1774	0.2668	0.2291
	SGS	0.1924	0.2505	0.2530
	SSSP	0.1977	0.2749	0.2576
	TANS	0.2042	0.2864	0.2673
	WJS	0.2023	0.2879	0.2645
	XWHS	0.2065	0.2929	0.2689

## Data Availability

The datasets analysed during the current study are available from the China National Center for Bioinformation/Beijing Institute of Genomics, Chinese Academy of Sciences repository, (https://ngdc.cncb.ac.cn/gvm/;GVM:.GVM000916, accessed on 20 July 2022). In addition, we downloaded 22 sheep breeds (PRJNA814428, PRJNA557060, and PRJNA624020) at the National Center for Biotechnology Information (NCBI) (https://www.ncbi.nlm.nih.gov/, accessed on 25 July 2022). Software parameters and code used in this article may be obtained by contacting the author if required.

## Supplementary Materials

The following supporting information can be downloaded at: https://www.mdpi.com/article/10.3390/ani15182747/s1, Table S1: Specific sampling locations for argali sheep; Table S2: Sources of all data tested in this paper; File S3: Methods employed during qPCR experiments; File S4: Treemix results file; File S5: ABBA-BABA results file; Table S6: GO and KEGG results file.

## Abbreviations

WGS	Whole Genome Sequencing
He	Expected Heterozygosity
Ho	Observed Heterozygosity
MAF	Allelic Frequency
π	Nucleotide Diversity
ROH	Runs of Homozygosity
LD	Linkage Disequilibrium
PCA	Principle Component Analysis
NJtree	Neighbor-Joining Tree
FST	Fixation Index
SNP	Single Nucleotide Polymorphism
GO	Gene Ontology
KEGG	Kyoto Encyclopedia of Genes and Genomes
qPCR	Quantitative Real-time PCR
