# Supplementary material for: Whole Genome Resequencing Reveals the Genetic Basis of Desert Arid Climate Adaptation in Lop Sheep
Source: Animals (Basel). 2025 Sep 19;15(18):2747. doi: 10.3390/ani15182747 (PMC12466512; doi:10.3390/ani15182747)
Supplement: Supplementary file 1 [file animals-15-02747-s001.zip › Supplementary File S3.pdf]

Supplementary Table S3

1. Main reagents and instruments

Table 1 main reagents and consumable

Reagent	Manufacturer
Trizol	Invitrogen
Chloroform	Tianjin Zhiyuan
Isopropyl alcohol	Tianjin Zhiyuan
Diethyl carbonate (DEPC)	Tiangen
Sodium hydroxide	Tianjin Zhiyuan
Reverse transcription kit	Shanghai Xinbei
Fluorescent quantitative kit	Shanghai Xinbei
Nucleic acid stain	Biotopped
Triethanolamine	Biotopped
Glacial acetic acid	Tianjin Beilian
Sodium ethylenediaminetetraacetate	Tianjin Fengchuan
PCRMix	Takara

Table 2 main instruments and equipment

Instrumentname	Manufacturer
-80°C ultra-low temperature refrigerator	Thermo Fisher Scientific
Handheld centrifuge	WEALTEC
Ice maker	xuehua
Adjustable micropipette	Eppendorf
Low-temperature high-speed refrigerated centrifuge	Sigma
Ultra-clean workbench	Boxun
PCR machine	BIORAD
Nucleic acid detector	Thermo Fisher Scientific
Fluorescence detector	Eppendorf
Gel imaging system	Bio-rad
Electrophoresis machine	Beijing Liuyi Biotechnology Co., Ltd.
Constant temperature water bath	Shanghai Boxun Medical Biological Instrument Co., Ltd.
Nanodrop One ultra-micro spectrophotometer	Thermo Fisher Scientific

2. Test method

2.1 Total RNA extraction from heart, liver, spleen, lung, and kidney tissues

of Lop sheep and Hu sheep

Total RNA was extracted from heart, liver, spleen, lung, and kidney tissues of Roebuck sheep and Lake sheep using the Trizol method. All materials used in the experiment were treated with RNase-free reagents.

(1) Add 1 ml of Trizol to the ground tissue, place it in a 1.5 ml enzyme-free EP tube, mix thoroughly, and let it stand for 5 minutes;

(2) Add 200 μL of chloroform to the enzyme-free centrifuge tube, shake vigor ously until pink, and let it sit on ice for 10 minutes;

(3) Centrifuge at 12,000 rpm at 4°C for 15 minutes, transfer approximately 40 0 μL of the supernatant to a 1.5 ml enzyme-free EP tube containing 400 μL o f isopropanol, gently invert to mix, and let it sit on ice for 10 minutes;

(4) Centrifuge at 12,000 rpm at 4°C for 10 minutes, discard the supernatant, a nd keep the white RNA precipitate at the bottom of the tube. Add 1 mL of p re-chilled 75% ethanol to wash the RNA precipitate, and gently invert to wash the precipitate.

(5) Centrifuge at 12,000 rpm at 4°C for 10 minutes, discard the ethanol, and r epeat the process of opening the lid and air-drying the white precipitate until i t becomes transparent. Add 30 μL of DEPC-treated water without RNAse to di ssolve the precipitate;

(6) Determine the RNA concentration using a nucleic acid concentration meter, perform 1% agarose gel electrophoresis to assess RNA quality, and store them at -80°C.

2.2 Reverse transcription to obtain the first strand of cDNA

The quality and concentration of total RNA were tested using a 1% agarose gel. Samples with an OD260/280 ratio between 1.8 and 2.0 were suitable for subsequent experiments. Reverse transcription was performed according to the instructions for the HyperScript III RT SuperMix for qPCR with gDNA Remover reverse transcription kit,

adding reagents as specified in Tables 3-3 and 3-4.

（1）gDNA removal system configuration and reaction procedure

Table 3 gDNA removal system

Reagent	Volume（μL）
Total RNA	5
8×gDNA remover	2
RNase-free ddH2O	9
Total volume	16

Table 4 Reverse transcription reaction system

Reagent	Volume（μL）
Step 1 Reaction liquid	16
5×RT SuperMix	4
Total volume	20

After adding the reagents, gently mix by pipetting and centrifuge for 1 minu te using a portable centrifuge. Place in a PCR machine and set the program to 37°C for 15 minutes, followed by 85°C for 5 seconds. Store the reverse trans cription products at -80°C.

3.3 Primer Design

Using bovine GAPDH (accession number NM_001034034.2) as the internal reference gene, qPCR detection was used to analyse the expression levels of the five candidate genes (PDGFD, NDUFS3, ATP1B2, ITGB8, and CD79A) selected in the signal analysis. Specific primers were designed based on RNA-seq sequences using Premier 6.0 software (synthesised by Shanghai Sangon Biotech Co., Ltd.), with primer information shown in Tables 3–5.

Table 5 Amplification primer information

Primer name	Primersequence	Annealing Temperature	Fragment length
PDGFD	F: CCAACCTCAGGCGAGATGAG<br>R: TGTGAGGTGATTGCTCTCAAGT	57	152
NDUFS3	F: GGTATGAGAGGGAGATCTGGGA<br>R: TCAACATAGCCAGACAGCGG	59	133
ATP1B2	F: ACCAGGGTTGATGATTCGCC<br>R: GGAGTCATTGTAAGGCTCCAAG	57	124
ITGB8	F: GCGGACTGCTTTGCATTATGT<br>R: TGCACATCTGTTGTCTTCACTT	57	161
CD79A	F: GAGAAGATGCCTGAGGGTCC<br>R: CCATGAGGAGTTGCCCAGG	59	96

3.4 qPCR reaction conditions

The qPCR reaction system is 10 μL: 5 μL of 2xS6 Universal SYBR qPCR Mix, 1 μL of cDNA template, 0.2 μL of each forward and reverse primer, and 3.6 μL of ddH_2O. qPCR reaction conditions: 95°C pre-denaturation for 30 seconds, 95°C denaturation for 5 seconds, 60°C annealing for 30 seconds, 72°C extension for 15 seconds, repeated for 40 cycles. Each sample is replicated three times.

3.5 qPCR data statistics and analysis

The relative expression levels of genes were calculated using the $2^{-\Delta\Delta Ct}$ method. One-way ANOVA was performed using SPSS 21.0, and GraphPad software was used for graphing. Note: ** represents highly significant (P<0.01); * represents significant (P<0.05); ns represents not significant (P>0.05).
