# Supplementary material for: Whole Genome Resequencing Reveals the Genetic Basis of Desert Arid Climate Adaptation in Lop Sheep
Source: Animals (Basel). 2025 Sep 19;15(18):2747. doi: 10.3390/ani15182747 (PMC12466512; doi:10.3390/ani15182747)
Supplement: Supplementary file 1 [file animals-15-02747-s001.zip › Supplementary Table S1.pdf]

# Supplementary Table S1

| Collection region | Number | Altitude（m） | annual rainfall（mm） | latitude | |
|---|---|---|---|---|---|
| Yuli County | 30 | 800 ~ 2782 | 43 | E: 84°02′∽89°58′ | N: 40°10′∽41°39′ |
| Ruoqiang County | 80 | 768 ~ 6900 | 28.5 | E: 86°45′ ~ 93°45′ | N: 36°05′ ~ 41°23′ |
